# Suggestions for resin research under the COST Action EU-PoTaRCh

**DOI:** 10.12688/openreseurope.18988.1

**Published:** 2024-12-20

**Authors:** Vítor João Pereira Domingues Martinho, Jakub Brózdowski

**Affiliations:** 1 Agricultural School (ESAV) and CERNAS-IPV Research Centre, Polytechnic Institute of Viseu, Viseu, Portugal; 2 Faculty of Forestry and Wood Technology, Poznań University of Life Sciences, Poznań, Poland

**Keywords:** Bibliometric analysis, Bibliometrix software, Gaps and Trends.

## Abstract

Forest management and planning is not an easy task due to, often, some intermittence in the incomes obtained by the landowners. In fact, namely in timber production, the producers must wait years to get revenues from the investments carried out on the lands they use. These frameworks become forest activities, in some circumstances, not attractive for the investors and, consequently, for adjusted management. The forest by-products appear as an opportunity to increase the profitability of the forest lands and motivate the land owners for more effective planning. This is crucial, namely in countries where forest fires, for example, are real problems for economic activity, populations and the environment. In this context, this study, developed within the scope of the COST Action PoTaRCh, intends to bring more insights and suggestions for the scientific research about resin. For that, a search was performed in the Scopus database (article title, abstract, keywords), on 02 November 2024, for the following topics: “natural resin” or “plant resin”. In this search, 4127 documents were obtained and assessed through bibliometric analysis. The findings identified reveal relevant gaps and trends in the literature that could be interesting outputs, particularly to support future research related to the different dimensions of resin worldwide.

## Introduction

The literature survey shows that, despite some efforts to apply bibliometric analysis in the fields associated with natural/plant resin ^1, 2^, there is still space to explore even more of the several dimensions related to this, often called, forest by-product (in a more reductive approach). Natural resin and gums are relevant complements of timber production in forest areas ^3^.

Bibliometric assessment is an approach that may produce relevant insights as support for the researchers, policymakers and other stakeholders in the diverse science fields, including in the forest domains, allowing to identification of gaps and trends in the literature ^4^. This is particularly important when, in some topics, we need to deal with a great number of documents to survey and understand what was already done and what we still need to do ^5^.

The literature also reveal also that some fields have been poorly addressed in the topics considered in this research, namely those associated with the following areas ^6^: social sciences; economics econometrics and finance; business, management and accounting; and decision sciences.

On the other hand, the areas related to “agricultural and biological sciences” have deserved a relevant attention of the researchers. Some of these studies give relevance to the resin synthesis by the trees ^7^ and others on the resin produced by bees (propolis) ^8^. The propolis is a resinous combination ^9^ that is obtained from plant resin ^10^ and used by the honeybees for their needs in beehives ^11^. This bee glue has several benefits for human health and has been used as an antioxidant or anti-inflammatory, for example ^12^. In some cases, propolis has been considered as a substitute for antibiotics ^13^, including in the poultry industry ^14^. Nonetheless, pests and pathogens, for example, create difficulties for the honeybee’s activities, where the role of the propolis still needs deeper analysis ^15, 16^.

Other research focuses associated with natural/plant resin are the following: extraction techniques, related economic dimensions and environmental impacts ^17^; tree response to wounding and fungi ^18^; Dragon’s Blood reddish resin oil ^19^; resin tapping ^20^; and the benefits of Chios mastic gum for human health ^21^.

Considering these perspectives, the aim of this study is to bring more insights into the diverse dimensions of natural/plant resin and make further suggestions for the work plan of COST Action CA22155 (EU-PoTaRCh) ^22^.

## Methods

This COST Action has as its scope the “Network for forest by-products charcoal, resin, tar, potash” and aims to analyse, over time, the productions that may be obtained in the forest lands, beyond the timber, highlighting chemical, biological, environmental, socioeconomic and cultural dimensions. EU-PoTaRCh is organised into five working groups focused on fields related to heritage, analytical approaches, archaeology, history and future scenarios ^22^. To achieve the objectives proposed in this research, 4127 documents were obtained from the Scopus database ^6^, in a search carried out on 02 November 2024 (for the topics: “natural resin” or “plant resin”), and were assessed through bibliometric analysis, following the procedures proposed by the Bibliometrix software ^23– 27^.

## Suggestions, gaps and trends from bibliometric analysis

For the study here presented, the period 1915–2024 was considered, for a total of 1742 sources, 4127 documents, 4.74% of annual growth rate, 20.59 average citations per document, 12538 authors, 3.96 co-authors per document and 12.41% of international co-authorships ( Table 1). Molecules, Contact Dermatitis, Phytochemistry, International Journal of Biological Macromolecules, Journal of Chromatography A, Fitoterapia, Food Chemistry and Journal of Agricultural and Food Chemistry are between the top 30 most important sources about the topics of natural resin or plant resin ( Table 2). Some of these sources are also among those with the highest impact within the sample considered ( Table 3). The most productive authors and those with the highest impact within the sample taken into account are presented in Table 4 and Table 5, respectively.

**Table 1.** Main information related to natural resin or plant resin.

Description	Results
Main Information about Data	
Timespan	1915:2024
Sources (Journals, Books, etc)	1742
Documents	4127
Annual Growth Rate %	4.74
Document Average Age	25.7
Average citations per doc	20.59
References	95836
Document Contents	
Keywords Plus (ID)	21866
Author's Keywords (DE)	6881
Authors	
Authors	12538
Authors of single-authored docs	626
Authors Collaboration	
Single-authored docs	687
Co-Authors per Doc	3.96
International co-authorships %	12.41
Document Types	
article	3663
book	2
book chapter	46
conference paper	108
conference review	2
data paper	4
editorial	11
erratum	4
letter	46
note	29
retracted	1
review	201
short survey	10

**Table 2.** Top 30 most relevant sources related to natural resin or plant resin.

Source	Articles
Molecules	97
Contact Dermatitis	65
Phytochemistry	45
International Journal of Biological Macromolecules	43
Journal of Chromatography A	42
Fitoterapia	40
Food Chemistry	40
Journal of Agricultural and Food Chemistry	37
Scientific Reports	34
Natural Product Research	33
Plos One	33
Journal of Natural Products	31
Journal of Ethnopharmacology	28
Meditsina Truda I Promyshlennaya Ekologiya	28
Gigiena i Sanitariia	26
Journal of Combinatorial Chemistry	26
Zhongguo Zhongyao Zazhi	25
Organic Letters	20
Journal of the American Chemical Society	19
Planta Medica	19
Journal of Natural Medicines	18
Analytical Biochemistry	17
International Journal of Molecular Sciences	17
Operative Dentistry	17
Analytical and Bioanalytical Chemistry	16
The Journal of Prosthetic Dentistry	16
Tree Physiology	16
Chemistry and Biodiversity	15
Biotechnic and Histochemistry	14
Journal of The American Dental Association	14

**Table 3.** Top 30 sources' local impact related to natural resin or plant resin.

Source	h_index
Molecules	26
Food Chemistry	23
Journal of Agricultural and Food Chemistry	23
Phytochemistry	21
Journal of Combinatorial Chemistry	19
Journal of Chromatography A	18
Fitoterapia	17
Journal of Natural Products	17
Contact Dermatitis	16
International Journal of Biological Macromolecules	16
Plos One	16
Journal of Ethnopharmacology	15
Tree Physiology	14
Organic Letters	13
Operative Dentistry	12
Phytomedicine	12
Planta Medica	12
Analytical and Bioanalytical Chemistry	11
Organic Geochemistry	11
Scientific Reports	11
Analytical Chemistry	10
Industrial Crops and Products	10
Journal of The American Chemical Society	10
Natural Product Research	10
Spectrochimica Acta - Part A: Molecular and Biomolecular Spectroscopy	10
The Journal of Prosthetic Dentistry	10
International Journal of Molecular Sciences	9
Journal of the American Dental Association	9
Analytical Biochemistry	8
Biomedicine and Pharmacotherapy	8

**Table 4.** Top 30 most relevant authors related to natural resin or plant resin.

Author	Articles
Li J	37
Wang X	25
Ono M	23
Zhang H	22
Chen Y	20
Kinjo J	20
Liu Y	20
Okawa M	20
Yoshimitsu H	20
Yasuda S	19
Wang J	18
Zhang J	18
Cheng Y-X	17
Chen X	16
Li Y	16
Pereda-Miranda R	16
Wang L	16
Zhang Y	16
Anderson KB	15
Song Z	15
Wang H	15
Wang Y	15
Al-Harrasi A	14
Chen H	14
Leonhardt SD	14
Nohara T	14
Spivak M	14
Zhang L	14
JR	13
Miyashita H	13

**Table 5.** Top 30 authors' local impact related to natural resin or plant resin.

Author	h_index
Li J	16
Wang X	14
Chen Y	13
Zhang H	13
Liu Y	12
Pereda-Miranda R	12
Spivak M	12
Chen H	11
Anderson KB	10
Chen X	10
Edwards Hgm	10
Leonhardt SD	10
Li K	10
Li Y	10
Song J	10
Wang C	10
Wang J	10
Wang L	10
Zhang J	10
Al-Harrasi A	9
Colombini MP	9
Lu Y	9
Merrifield RB	9
Wang H	9
Zhang Y	9
Cheng Y-X	8
Kinjo J	8
Liu X	8
Nohara T	8
Okawa M	8

The most important affiliations are the following ( Table 6): Tokai University; Beijing University of Chinese Medicine; National and Kapodistrian University of Athens; Chinese Academy of Medical Sciences and Peking Union Medical College; University of Nizwa; Sojo University; Fukuoka University; and China Pharmaceutical University. China, USA, India, Brazil, Japan, Italy, Germany, UK, France, Iran, Greece and Spain are the most productive countries ( Table 7) and are among the most cited nations ( Table 8).

**Table 6.** Top 30 most relevant affiliations related to natural resin or plant resin.

Affiliation	Articles
Tokai University	111
Beijing University of Chinese Medicine	90
National and Kapodistrian University of Athens	62
Chinese Academy of Medical Sciences and Peking Union Medical College	59
University of Nizwa	59
Sojo University	55
Fukuoka University	54
China Pharmaceutical University	53
Nanjing University of Chinese Medicine	53
Northwest Aandf University	53
Shandong University	51
Nanjing Forestry University	50
Guangdong Pharmaceutical University	49
University of Alberta	48
Institute of Chemical Industry of Forest Products	46
Shenyang Pharmaceutical University	46
University of São Paulo	46
King Saud University	43
Peking Union Medical College	43
Universidad Nacional Autónoma de México	42
Jiangnan University	40
Guangxi University for Nationalities	39
Silpakorn University	39
Notreported	38
University of Messina	38
Mashhad University of Medical Sciences	35
University of Minnesota	34
Aristotle University of Thessaloniki	32
Nantong University	31
Shenzhen University Health Science Center	31

**Table 7.** Top 30 countries' scientific production related to natural resin or plant resin.

Country	Number of documents
China	2830
USA	1728
India	989
Brazil	885
Japan	674
Italy	595
Germany	555
UK	481
France	386
Iran	270
Greece	251
Spain	249
Canada	225
Egypt	224
Mexico	214
Poland	189
South Korea	178
Australia	168
Thailand	163
Turkey	161
Saudi Arabia	144
Finland	143
Romania	140
Switzerland	130
Belgium	118
Sweden	117
Austria	114
Chile	99
Indonesia	90
Pakistan	83

**Table 8.** Top 30 most cited countries related to natural resin or plant resin.

Country	Total Citations
China	7512
USA	6685
India	3645
Brazil	3555
Germany	2837
Italy	2465
Malaysia	1498
France	1492
United Kingdom	1277
Austria	1178
Canada	1149
Japan	1112
Egypt	1055
Iran	1032
Greece	889
Australia	876
Switzerland	701
Thailand	671
Korea	661
Mexico	595
Spain	554
Turkey	531
Finland	483
Denmark	472
Poland	430
Chile	343
Singapore	302
Belgium	297
Oman	292
Israel	284

**Figure 1.**
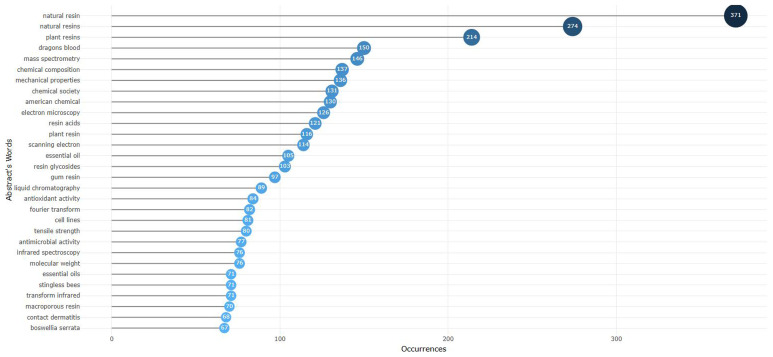
Most frequent words (in abstracts) related to natural resin or plant resin.

**Figure 2.**
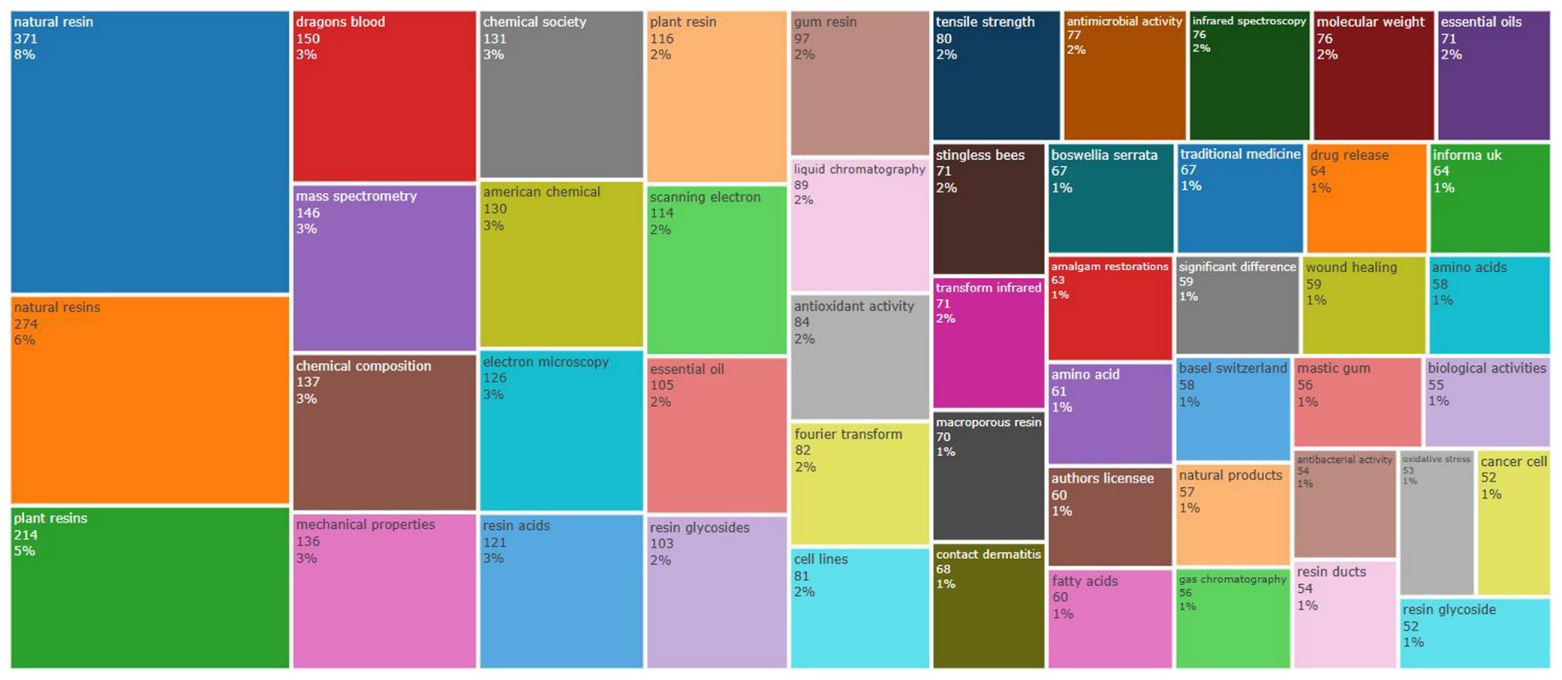
TreeMap (in abstracts) related to natural resin or plant resin.

Beyond the topics of search considered in this study (natural resin and plant resin), dragon’s blood and mass spectrometry are examples of the most frequent words in the abstracts of the 4127 documents taken into account in this analysis ( Figure 1). Other words appear with lower frequency, such as ( Figure 2): amalgam restorations; fatty acids; traditional medicine; natural products; wound healing; mastic gum; antibacterial activity; resin ducts; biological activities; oxidative stress; and resin glycoside.

**Figure 3.**
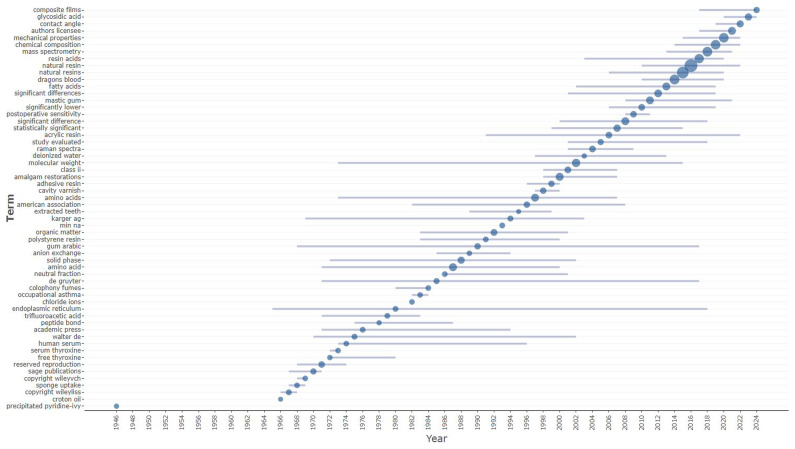
Trend topics (in abstracts) related to natural resin or plant resin.

**Figure 4.**
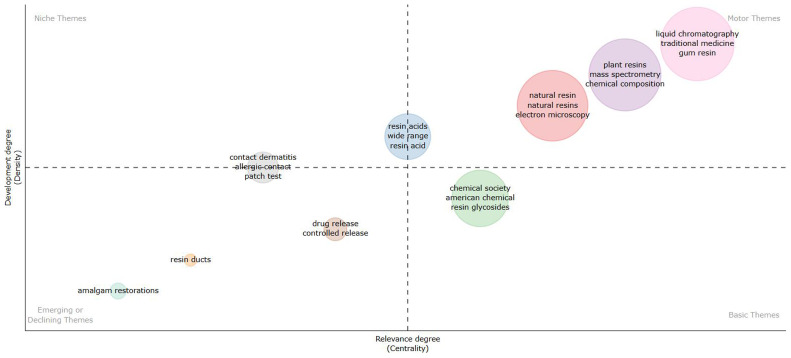
Thematic map related to natural resin or plant resin.

**Figure 5.**
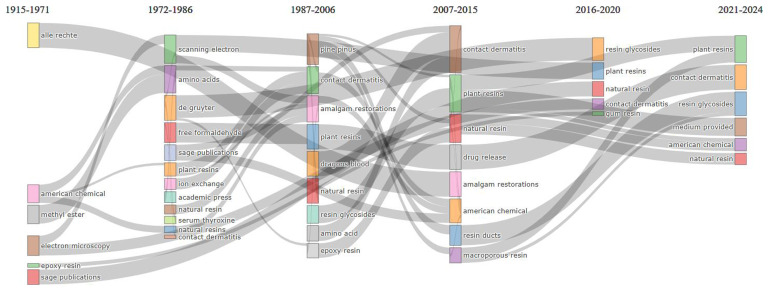
Thematic evolution related to natural resin or plant resin.

**Figure 6.**
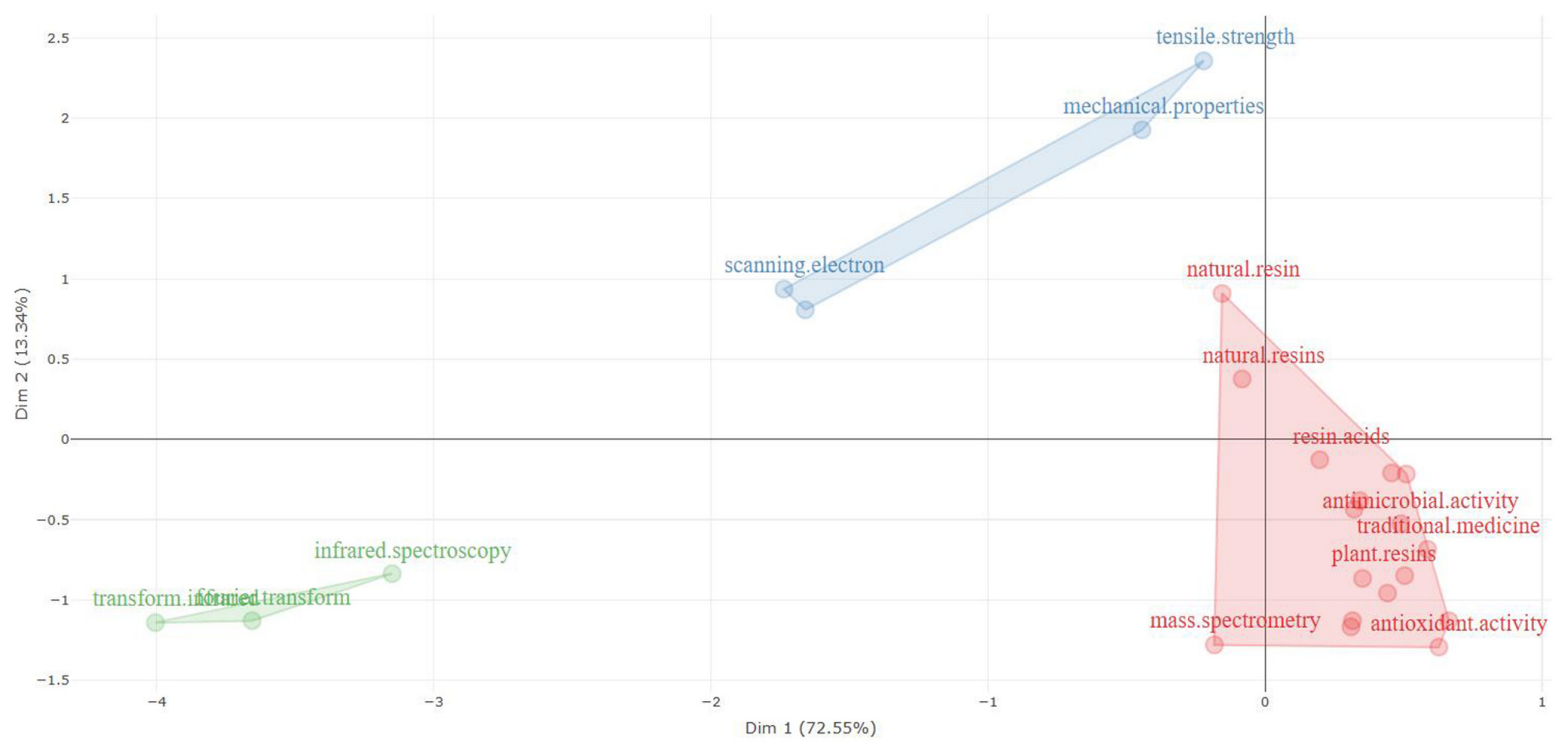
Factorial analysis related to natural resin or plant resin.

On the other hand, composite films, glycosidic acid, mechanical properties, chemical composition, mass spectrometry, resin acids, natural resin, dragon’s blood, fatty acids and mastic gum are trending terms ( Figure 3). Figure 4 shows (considering information in abstracts and Walktrap clustering algorithm) that amalgam restorations, resin ducts, drug release and controlled release are emerging or declining themes. Resin glycosides is an important theme, but not yet well developed. Electron microscopy, mass spectrometry, chemical composition, traditional medicine and gum resin are important and well-developed themes. Figure 5 (taking into account information in abstracts, Walktrap as clustering algorithm and inclusion index weighted by word-occurrences as weight index) confirms that plant resin and natural resin are trending themes. Figure 6 and Table 9 (factorial analysis in abstracts and multiple correspondence analysis) highlight three clusters for the words present in the abstracts and show that the words considered in cluster 1 are central for the research of the topics here addressed, considering the documents surveyed.

**Table 9.** Words by cluster related to natural resin or plant resin.

word	cluster
natural.resin	1
natural.resins	1
plant.resins	1
dragons.blood	1
mass.spectrometry	1
chemical.composition	1
resin.acids	1
plant.resin	1
essential.oil	1
resin.glycosides	1
gum.resin	1
liquid.chromatography	1
antioxidant.activity	1
cell.lines	1
antimicrobial.activity	1
traditional.medicine	1
biological.activities	1
mechanical.properties	2
electron.microscopy	2
scanning.electron	2
tensile.strength	2
fourier.transform	3
infrared.spectroscopy	3
transform.infrared	3

## Discussion and conclusions

This research was developed under the work plan of the COST Action PoTaRCh and aims to bring more contributions to the understanding of the several dimensions related to resin and presents suggestions for future studies associated with these topics. To achieve these objectives, a bibliometric analysis was carried out, following the procedures proposed by the Bibliometrix software. These documents were obtained from the Scopus database, in a search performed on 02 November 2024, for the topics “natural resin” or “plant resin”.

The literature highlights the importance of the natural/plant resin for forest management, considering that this by-product may be an interesting complement of the forest owners’ income. The consideration of alternative and sustainable sources of revenues on forest land, beyond wood production, is crucial to motivate more adjusted forestry planning, namely in contexts where such adjustments are needed and urgent. This is particularly indispensable in frameworks where forest fire prevention requires innovative and effective approaches. The bibliometric analysis may bring important highlights in the literature survey on any topic, including in the fields related to the forest dimensions. On the specific topics covered here, the literature survey reveals that the scientific areas associated with social sciences, economics and management may be further explored in future research. In turn, the areas associated with agricultural and biological sciences have been addressed by a relevant number of documents. In these studies, a relevant part considered the different dimensions of the propolis (resinous combination produced by bees from plant resin). Other studies analysed extraction techniques, tree response to external aggressions, dragon’s blood resin and resin tapping, for example.

The bibliometric assessment highlights a modest international co-authorship of 12.41% and that the most important sources (number of documents and citations) are related, for example, to contact dermatitis, phytochemistry, biological macromolecules, chromatography and food chemistry. This shows that there is shows that there is space to be explored in other sources with scopes more multidisciplinary and focused on other scientific areas. The most productive countries, the topics here addressed, are China, the USA, India and Brazil, revealing that some European countries, for example, where the natural/plant resin has a historical relevance may add important contributions to these fields. The bibliometric analysis also shows that natural resin, plant resin, dragon’s blood, mass spectrometry, chemical composition, resin acids, essential oils, resin glycosides, gum resin, antioxidant activity and traditional medicine are central topics for the topics natural/plant resin. Some of them are also trending topics, such as chemical composition, mass spectrometry, resin acids, natural resin and dragon’s blood.

In terms of practical implications, this research highlights trending themes related to natural/plant resin topics and that some scientific areas, such as social sciences, economics and management, could be further addressed by the literature. In terms of policy recommendations, it is suggested to promote more transdisciplinary approaches in the analyses related to the different dimensions of resin production and collection, to better support the various stakeholders in managing and planning the natural/plant resin activities. For future research, it would be important to increase international co-authorships, promote transdisciplinary co-authorships and consider other bibliometric methodologies.

## Data Availability

No data are associated with this article
