# Supplementary material for: Suggestions for resin research under the COST Action EU-PoTaRCh
Source: Open Res Eur. 2025 Aug 4;4:272. Originally published 2024 Dec 20. [Version 2] doi: 10.12688/openreseurope.18988.2 (PMC12340490; doi:10.12688/openreseurope.18988.2)
Supplement: Peer review history

# Reviewer report: Reviewer response for version 2

**DOI:** 10.21956/openreseurope.22834.r57453

**Published:** 2025-08-18

**Authors:** Marco Biagi

**Affiliations:** 1 University of Parma, Parma, Italy

I'd want to thank authors for the revision which resulted in an overall improvement of the manuscript, with clearer presentation and better organization of the data.

 However, as all reviewers consistently pointed out, a key structural weakness remained, not fully fixed: the connection between the stated aim, aligned with the broader objectives of COST Action CA22155 (EU-PoTaRCh), and the bibliometric analysis conducted is still not fully developed. The paper effectively describes the state of research and identifies significant gaps, but yet it does not translate these findings into operational perspectives or concrete strategies that could guide future action. In light of the Action mission, I recommend authors an additional effort to transform the bibliometric evidence into targeted, integrated perspectives, strengthening the paper as a decision-making tool rather than solely a mapping document.

 As regards this point, I suggest to include a dedicated and extensive section on limitations and future perspectives, explicitly discussing methodological constraints, potential biases in data collection and outlining how future research could address the identified gaps. This section could also provide examples of actions, including how to foster cross-disciplinary collaborations in underrepresented fields or strategies for stimulating research, thereby increasing the paper practical value for stakeholders and policy makers.

Is the argument persuasive and supported by appropriate evidence?

No

Is the work clearly and cogently presented?

No

Is the topic of the essay discussed accurately in the context of the current literature?

Partly

Does the essay contribute to the cultural, historical, social understanding of the field?

Partly

Reviewer Expertise:

Pharmaceutical biology, pharmacognosy, phytochemistry, phytotherapy

I confirm that I have read this submission and believe that I have an appropriate level of expertise to confirm that it is of an acceptable scientific standard, however I have significant reservations, as outlined above.

# Reviewer report: Reviewer response for version 1

**DOI:** 10.21956/openreseurope.20548.r56657

**Published:** 2025-08-11

**Authors:** İnci Caglayan

**Affiliations:** 1 Istanbul University-Cerrahpaşa, Sarıyer, Turkey

This study aims to provide a bibliometric analysis on natural and plant resins within the scope of the COST Action EU-PoTaRCh. While the topic is timely and relevant, the manuscript has several shortcomings regarding structure, originality, and interpretation. The paper relies heavily on basic bibliometric outputs and lacks sufficient analysis and discussion. Below are my specific comments:

 1-Abstract: The abstract is not clear enough. It does not explain the aim, method, or key findings well. The contribution of the study is also not obvious. A clearer and more structured abstract is needed.

 2-The authors present this study as a bibliometric analysis. However, it would benefit from clarifying whether the approach follows a thematic or chronological perspective. Currently, it reads more like a general listing of publication metrics rather than a structured review with clear categories or insights. Bibliometric analysis is a tool—not a review type.

 3-The introduction does not clearly follow a logical structure. It should explain (1) why the topic is important, (2) what the research gap is, and (3) how this study addresses that gap. Currently, these points are not well developed.

 4-The data presented in Tables 1 to 8 appear to be standard outputs from Scopus or Web of Science. These are descriptive and not supported by further interpretation or original evaluation. To enhance the value of the study, the authors should provide more critical insight or thematic classification based on these results.

 5- Figure 2 is a standard tree map that can be directly generated by Scopus or Web of Science. It lacks added interpretation or connection to the research objectives. The authors should go beyond visual representation and provide analytical insights.

 6- The manuscript reports bibliometric indicators (e.g., citation counts, co-authorships), but it does not explain what these mean in terms of research development or scientific progress in resin-related studies.

 7-There is excessive focus on propolis and bee-related resins, which may not fully align with the scope of forest-based resin production unless justified more clearly.

 I believe the manuscript has potential, but it requires **major revisions** before being considered for indexing.

Is the argument persuasive and supported by appropriate evidence?

Partly

Is the work clearly and cogently presented?

Partly

Is the topic of the essay discussed accurately in the context of the current literature?

Partly

Does the essay contribute to the cultural, historical, social understanding of the field?

Yes

Reviewer Expertise:

I specialize in forest planning and have conducted several taxonomic and thematic literature reviews, particularly on forest management, wildfires, and species distribution modeling. I also have experience with literature reviews and forest-based ecosystem services such as resin production. Based on this background, I was able to assess the article in terms of its structure, methodology, literature coverage, and contribution to research gaps. My review focuses specifically on the literature review approach and its implementation in the study.

I confirm that I have read this submission and believe that I have an appropriate level of expertise to confirm that it is of an acceptable scientific standard, however I have significant reservations, as outlined above.

# Reviewer report: Reviewer response for version 1

**DOI:** 10.21956/openreseurope.20548.r56660

**Published:** 2025-07-28

**Authors:** Lilia Ortiz Rodríguez

**Affiliations:** 1 Tecnológico Nacional de México/ Instituto Tecnológico Superior de Perote, Veracruz, Mexico

The text discusses the topic of forest resin with notable precision. It presents a solid literature review that enables an understanding of the current state of knowledge in this field, clearly identifying both the areas that have received scholarly attention and those that still require further investigation. The information is presented in a clear and organized manner, which facilitates comprehension even when diverse topics are addressed. The thematic progression is well structured. Although technical concepts are used, the overall organization and language make the content accessible to academic and professional readers.

 The text does not employ overtly persuasive language; however, it effectively convinces the reader of the importance of bibliometric analysis in this field. The identification of thematic gaps and the proposal of new research directions serve as strong arguments to justify the continuation of the study and the need for initiatives such as the COST Action PoTaRCh.

 The text also provides elements that contribute indirectly to the cultural, historical, and social understanding of resin use. By referencing traditional practices such as resin harvesting and the use of propolis, it opens the door to analysis from other disciplinary perspectives. Furthermore, by highlighting the lack of research in social and economic areas, it encourages the integration of these approaches to achieve a more comprehensive understanding of the topic.

Is the argument persuasive and supported by appropriate evidence?

Yes

Is the work clearly and cogently presented?

Partly

Is the topic of the essay discussed accurately in the context of the current literature?

Yes

Does the essay contribute to the cultural, historical, social understanding of the field?

Yes

Reviewer Expertise:

My area of specialization lies in agronomic and forest sciences. Last year, I carried out a funded project focused on the application of forest resin as a biofilm in food products. Therefore, I consider this work to be highly impactful, as further interdisciplinary research is still needed to advance the study of resin as a forest by-product with the potential to be sustainable and to contribute to a circular economy model that benefits local communities.

I confirm that I have read this submission and believe that I have an appropriate level of expertise to confirm that it is of an acceptable scientific standard.

# Reviewer report: Reviewer response for version 1

**DOI:** 10.21956/openreseurope.20548.r56658

**Published:** 2025-07-25

**Authors:** M. V. Velasco-García

**Affiliations:** 1 CENID COMEF, National Institute of Forestry, Agriculture and Livestock Research (INIFAP), Ciudad de México, Coyoacán, Mexico

The bibliometric study *"Suggestions for Resin Research under the COST Action EUPoTaRCh"* aims to assess the scientific output related to natural resins. The objective of the research was to provide insight into the various dimensions of natural/plant-based resins and to offer suggestions for the CA22155 Work Plan under the COST Action (EU-PoTaRCh). The study generates valuable information on the most relevant sources on resins and their impact, as well as on the influence and affiliations of key authors, the countries with the highest scientific output and their citation rates, the most frequent keywords associated with natural resins, and the thematic evolution and clustering of the topic. All of this constitutes the main strengths of the study.

 In contrast, the presentation of the study reveals several weaknesses:

- **The introduction is weak.** It lacks sufficient background and fails to highlight the relevance and importance of the topic, as well as a clear articulation of the research problem. Additionally, there is an absence of clearly defined research questions or hypotheses guiding the investigation.
- **The results section** requires improvement in its presentation. In several parts of the text, information is redundantly presented in both the narrative and the tables.
- **The discussion and conclusions are deficient.** Certain paragraphs—or parts of them—do not correspond to a critical analysis of the results. For example, the first paragraph focuses on the study’s objective in the context of the EU-PoTaRCh COST Action work plan, which would be more appropriate in the introduction. The latter part of that paragraph seems more aligned with the methodology section. Much of the second paragraph follows the same pattern. The remainder of the discussion is too brief considering the extensive results presented; a deeper analysis is needed to explore the underlying causes and implications of the findings for the present and future of various dimensions of natural resin research. Moreover, this section should have included a detailed discussion of the content of the publications reviewed—specifically, how resin-related research has contributed to the advancement of the field. A particularly notable omission is the absence of concrete “suggestions for the CA22155 Work Plan of the COST Action (EU-PoTaRCh),” as proposed in the second part of the research objective. Furthermore, this section fails to explicitly present the main conclusions of the study, which should be clearly articulated based on the research objectives, hypotheses or questions, and key findings.

In summary, the study offers robust and extensive findings regarding the multiple dimensions of natural resin research, and serves as a solid foundation for guiding future investigations. However, substantial revision is required across all sections, particularly in the discussion, where a more thorough interpretation of the results is needed to ensure alignment with the stated research objectives.

Is the argument persuasive and supported by appropriate evidence?

Partly

Is the work clearly and cogently presented?

No

Is the topic of the essay discussed accurately in the context of the current literature?

Partly

Does the essay contribute to the cultural, historical, social understanding of the field?

Partly

Reviewer Expertise:

Selection and genetic improvement of resin species

I confirm that I have read this submission and believe that I have an appropriate level of expertise to confirm that it is of an acceptable scientific standard, however I have significant reservations, as outlined above.

# Reviewer report: Reviewer response for version 1

**DOI:** 10.21956/openreseurope.20548.r49526

**Published:** 2025-01-23

**Authors:** Marco Biagi

**Affiliations:** 1 University of Parma, Parma, Italy

The study titled "Suggestions for Resin Research under the COST Action EU-PoTaRCh" aims to provide with valuable insights into the various dimensions of natural resins through a bibliometric analysis. The goal to identify gaps and trends in resin-related research and provide suggestions for future studies is timely and relevant, given the increasing interest in sustainable forest by-products and their socioeconomic and environmental implications.

 In my opinion, the study has strenghts and limitations, detailed below.

 Strengths of the Study.

 The authors effectively highlight the under-representation of specific research areas, particularly in social sciences, economics, and management.

 The paper provides relevant suggestions on the need for transdisciplinary approaches for the study and exploitation of resins.

 Despite these strengths, the study presents several limitations that need to be addressed to enhance its overall impact and clarity.

 While the paper sets out to explore the multiple dimensions of resin (chemical, biological, environmental, socioeconomic, and cultural), the bibliometric methodology employed is not sufficiently linked to these objectives.

 The focus is primarily on quantitative metrics such as publication count, citations, and authorship, which do not directly address the stated multidimensional aspects of resin. The study does not sufficiently analyze the content of the publications to explain how resin research has evolved across different disciplines. In other terms, the study lacks an in-depth thematic discussion that connects the identified items with different topics and applications.

 The discussion does not adequately link findings to concrete policy recommendations that stakeholders could adopt.

 Overall, the study provides a solid foundation for further research on resin; however, major revisions and improvements are needed in aligning the methodology with the objectives and enhancing the interpretation of the results.

Is the argument persuasive and supported by appropriate evidence?

No

Is the work clearly and cogently presented?

No

Is the topic of the essay discussed accurately in the context of the current literature?

Partly

Does the essay contribute to the cultural, historical, social understanding of the field?

Partly

Reviewer Expertise:

Pharmaceutical biology, pharmacognosy, phytochemistry, phytotherapy

I confirm that I have read this submission and believe that I have an appropriate level of expertise to state that I do not consider it to be of an acceptable scientific standard, for reasons outlined above.
